# Contained morcellation of the spleen using a completely enclosed laparoscopic isolation system: a 5-year follow-up study on the evaluation of splenosis

**DOI:** 10.3389/fsurg.2026.1876074

**Published:** 2026-08-11

**Authors:** Chang Zhou, Wenhui Wang, Li Xu, Shiyu Zhang, Jing Liang, Zhiying Yang, Bin Ling

**Affiliations:** 1 Chinese Academy of Medical Sciences & Peking Union Medical College, Beijing, China; 2 Department of Obstetrics and Gynecology, China-Japan Friendship Hospital, Beijing, China; 3 Department of Gynecologic Oncology, Beijing Obstetrics and Gynecology Hospital, Capital Medical University, Beijing Maternal and Child Health Care Hospital, Beijing, China; 4 Department of Hepatobiliary Surgery, China-Japan Friendship Hospital, Beijing, China

**Keywords:** contained morcellation, isolation system, laparoscopic splenectomy, splenomegaly, splenosis

## Abstract

**Background:**

Specimen extraction in massive splenomegaly remains a major challenge in laparoscopic splenectomy. To address this, the Completely Enclosed Laparoscopic Isolation System (CELIS) was utilized to enable contained morcellation without incision enlargement.

**Methods:**

This single-arm retrospective study included 14 patients undergoing CELIS-assisted laparoscopic splenectomy with fully enclosed morcellation under intrabag pneumoperitoneum.

**Results:**

All procedures were completed without conversion or incision enlargement. Mean operative time was 182.86 ± 52.84 min, with efficient CELIS deployment and morcellation. Postoperative pain was low (VAS 2.64 on day 1), and cosmetic outcomes were favorable. Within this small, single-arm retrospective cohort (*n* = 14), 0/14 cases of clinically or radiologically detectable splenosis or ectopic splenic implantation were observed during the 5-year follow-up (95% CI: 0.0%–23.2%), suggesting the preliminary safety and feasibility of this technique.

**Conclusions:**

This preliminary study suggests that CELIS-assisted contained morcellation is technically feasible for large to massive spleens. The 5-year follow-up provides preliminary observational data regarding the absence of detectable splenosis in this selected cohort, though larger comparative studies are required.

## 1 Introduction

The widespread adoption of laparoscopic techniques represents a major milestone in modern surgery, shifting the paradigm from large incisions to minimally invasive precision. However, when managing massive solid organs, a critical limitation persists—namely, the paradox of “minimally invasive resection but traumatic specimen extraction.”

Laparoscopic splenectomy (LS), first introduced in 1991, has become a well-established procedure (1). With advances in imaging and energy devices, even massive spleens can be safely mobilized laparoscopically. Nevertheless, once the spleen is completely dissected, extraction through a 10–12 mm trocar remains a formidable challenge. Conventional approaches often require extension of the incision or creation of an accessory incision, which negates the fundamental benefits of minimally invasive surgery, including reduced postoperative pain, lower risk of incisional hernia, and improved cosmetic outcomes (2).

To preserve the integrity of small-incision surgery, power morcellation has been introduced as a potential solution (3). However, the spleen is highly vascular, friable, and possesses strong regenerative capacity (4, 5). Performing uncontained morcellation within the open abdominal cavity poses a substantial risk. High-speed rotating blades can disperse splenic fragments throughout the peritoneal cavity, leading to iatrogenic implantation of splenic tissue, known as splenosis (4).

Splenosis is a well-recognized phenomenon following splenic rupture or fragmentation, in which viable splenic tissue implants and proliferates within the peritoneal cavity (6). These ectopic implants may cause chronic abdominal pain, bowel obstruction, or be mistaken for malignancy on imaging, resulting in unnecessary interventions.

Importantly, concerns regarding tissue dissemination are not merely theoretical. The U.S. Food and Drug Administration (FDA) has issued multiple safety communications and black box warnings regarding the use of power morcellation, emphasizing that uncontained morcellation may spread both malignant and benign tissues within the abdominal and pelvic cavities (7, 8). These warnings underscore the critical need for effective containment strategies during tissue fragmentation.

Given this unmet clinical need, we hypothesized that the key to improving minimally invasive management of large specimens lies not in abandoning morcellation, but in achieving complete physical isolation. To this end, the CELIS was designed to create an independent, sealed working chamber within the abdominal cavity. This system allows controlled, visualized morcellation under reliable containment.

This study presents the surgical technique and reports the 5-year follow-up outcomes of CELIS-assisted laparoscopic splenectomy, exploring its preliminary clinical feasibility and long-term clinical outcomes profile regarding tissue dissemination in a limited cohort.

## 2 Materials and methods

### 2.1 Study design and population

This single-arm retrospective cohort study enrolled 14 patients who underwent laparoscopic splenectomy (LS), including multiple cases of massive splenomegaly, combined with fully closed power morcellation under the protection of the CELIS system at China-Japan Friendship Hospital between September 2020 and March 2021. All surgeries were performed by the same senior surgical team (Z.Y. and J.L.) with rigorous training in minimally invasive techniques.

All procedures in this study were conducted in strict accordance with the ethical standards of the national research committee and the 1964 Declaration of Helsinki and its later amendments. The study protocol was officially approved by the Institutional Review Board (IRB) of China-Japan Friendship Hospital. Written informed consent was obtained from all patients prior to surgery.

The inclusion criteria were: (1) patients aged 18–75 years; (2) symptomatic or massive splenomegaly requiring laparoscopic splenectomy; and (3) confirmed benign splenic lesions or non-malignant hematological disorders (e.g., severe hypersplenism secondary to portal hypertension). The exclusion criteria were: (1) suspected or pathologically confirmed malignant hematological diseases (such as lymphoma or leukemia) where an intact spleen is mandatory for definitive pathological staging and diagnosis; (2) history of extensive upper abdominal surgeries leading to severe adhesions; (3) acute splenic rupture with hemodynamic instability; and (4) severe coagulopathy uncorrectable prior to surgery. Furthermore, none of the enrolled patients presented any contraindications for tissue morcellation. Preoperative exclusion of malignancy was based on standard clinical workup, including medical history, routine blood tests, and tumor markers. Contrast-enhanced imaging (CT or MRI) was routinely evaluated to identify typical features of benign lesions (e.g., hemangiomas) or to confirm secondary causes of splenomegaly, such as portal hypertension. In cases where primary hematologic malignancy could not be entirely excluded by initial screening, further evaluations, including hematology consultation and peripheral blood smear analysis, were conducted prior to surgery.

### 2.2 Device description

**Figure 1:**
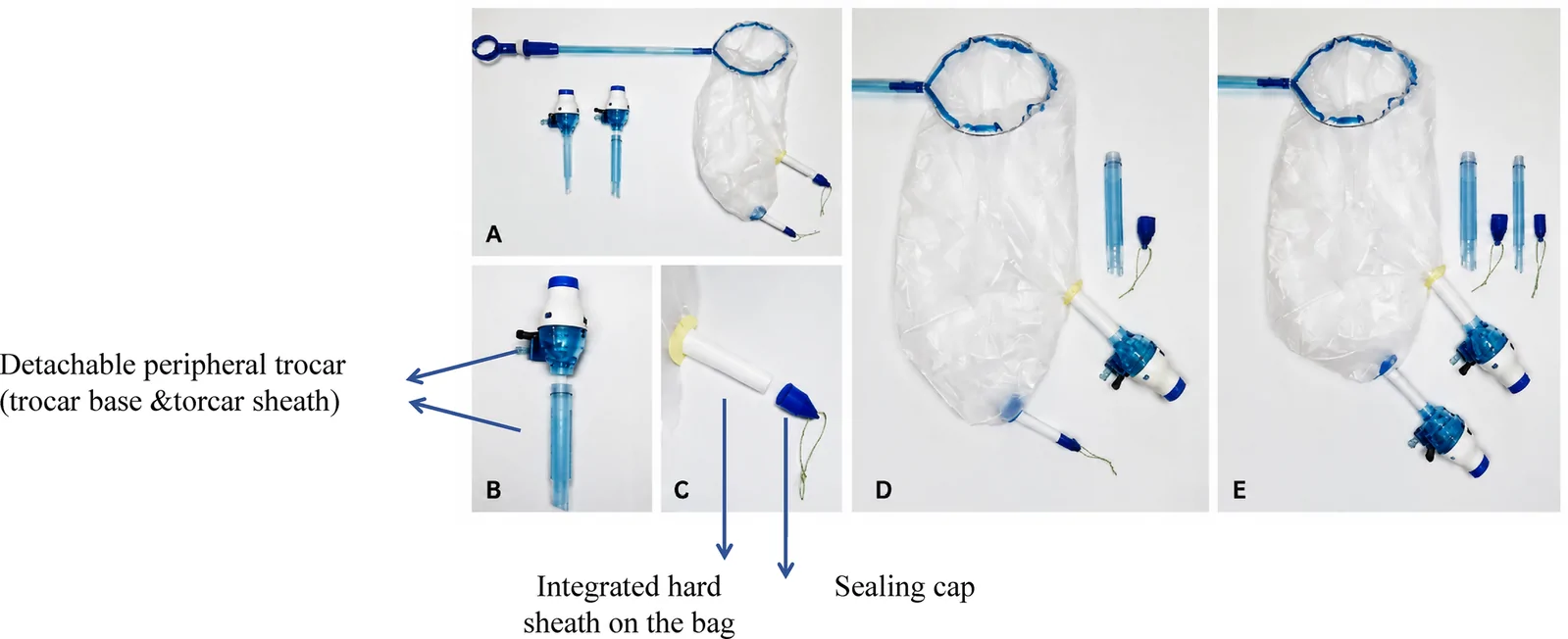
Main components of the multi-port containment (CELIS) system. **(A)** The main components of the CELIS system include the detachable trocar and the retrieval bag. **(B)** Detachable trocar (trocar base and trocar sheath). **(C)** Hard sheath on the bag and sealing cap. **(D,E)** Transposing sealing caps and trocar bases by threaded structures. Reprinted with permission from (17).

The CELIS system (Figures 1A–E) is a sterile, single-use disposable medical device that has received official regulatory approval from the National Medical Products Administration (NMPA) of China (Registration No. 20192020101) and is commercially manufactured by Hefei Hebo Medical Device Co., Ltd. (Hefei, China). It consists of two core components: (1) a tear-resistant, transparent, multi-port isolation bag manufactured from medical-grade polymer, featuring integrated hard sheaths; and (2) detachable peripheral trocars, each comprising a trocar base and a trocar sheath (Figure 1B). To accommodate massive splenic specimens, the isolation bag features a large opening diameter of 100 mm and a maximum volumetric capacity of 3,500 mL.

A key design feature of the CELIS system is its interchangeable airtight sealing mechanism (Figures 1C–E). The integrated hard sheaths on the isolation bag are initially closed by tethered, threaded sealing caps (Figure 1C) to maintain a reliable seal. During the procedure, these sealing caps can be removed, and the detachable trocar bases can be rotated and securely screwed directly onto the bag's integrated hard sheaths (Figures 1D,E). Because these rotating trocar bases are equipped with standard gas valves and sealing membranes, they effectively establish a secure intrabag pneumoperitoneum. More importantly, this design ensures that the bag's working channels are fully compatible with conventional laparoscopic equipment (including insufflation tubing and laparoscopes) as well as standard 12–15 mm electromechanical power morcellators.

### 2.3 Surgical procedures

#### 2.3.1 Patient positioning

Under general anesthesia, patients were placed in a 30-degree right lateral decubitus position.

#### 2.3.2 Trocar placement

**Figure 2:**
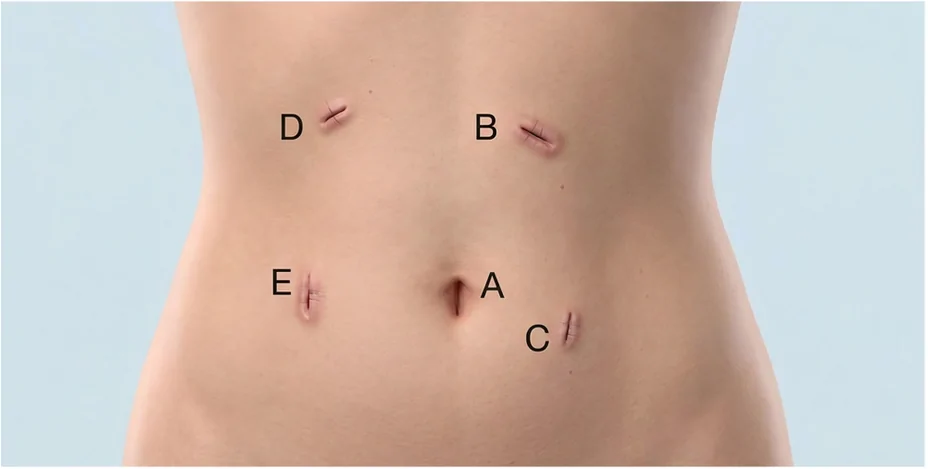
Schematic illustration of the trocar positions. This is a computer-generated graphic created using Adobe Illustrator and does not depict a real patient. (A) 10-mm observation port at the superior umbilical margin. Three 5-mm working ports located (B) in the left hypochondrium, (C) 5 cm below the left paraumbilical line, (D) 3 cm below the right hypochondrium-midclavicular intersection, and (E) 12-mm main working port 5 cm above the right paraumbilical line. Pneumoperitoneum was maintained at 12–14 mmHg.

A classic five-port technique was utilized (Figure 2). A 10-mm observation port was placed at the superior margin of the umbilicus (Point A). Three 5-mm working ports were placed in the left hypochondrium (Point B), 5 cm below the left paraumbilical line (Point C), and 3 cm below the intersection of the right hypochondrium and the right midclavicular line (Point D). A 12-mm main working port was placed 5 cm above the right paraumbilical line (Point E). Conventional pneumoperitoneum pressure was maintained at 12–14 mmHg.

#### 2.3.3 Splenic mobilization

The enlarged spleen was fully mobilized following standardized procedures, which included meticulous dissection and transection of the perisplenic ligaments and splenic hilar vessels.

#### 2.3.4 CELIS system insertion and intrabag pneumoperitoneum establishment (video 1)

(a) The CELIS system was smoothly inserted into the abdominal cavity via Point E (12-mm trocar sheath). (b) The highly resilient isolation bag was unfolded, and the mobilized enlarged spleen was pushed into the bag. (c) The opening edge of the isolation bag was extracted extracorporeally via Point E using non-traumatic graspers. (d) The integrated rigid working sheaths of the isolation bag were drawn out of the abdominal wall through their corresponding trocar sites (the large rigid sheath via Point A, and the small rigid sheath via Point C). (e) The sealing caps of the rigid sheaths were replaced with trocar bases connected to the laparoscopic insufflator. (f) Core Procedure: The original abdominal pneumoperitoneum was completely deflated. Instead, CO₂ gas was insufflated into the sealed isolation bag via the trocar to establish an “independent intrabag pneumoperitoneum” (pressure 12–14 mmHg). The isolation bag expanded like a balloon, completely displacing the enlarged spleen from surrounding intestines and organs, thereby achieving reliable physical isolation.

#### 2.3.5 Fully closed direct-vision power morcellation (video 1)

**Figure 3:**
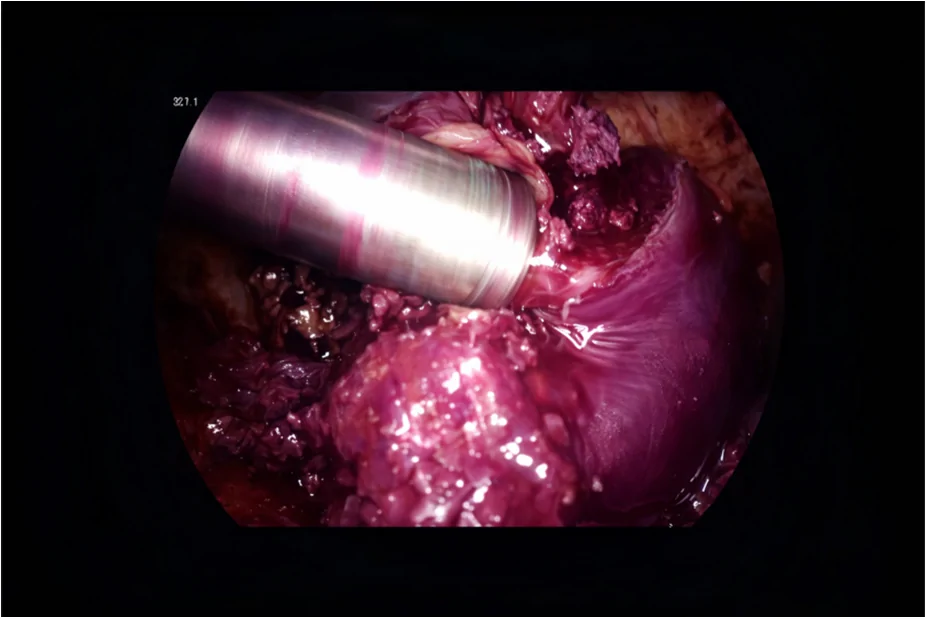
Power morcellation in CELIS system.

**Figure 4:**
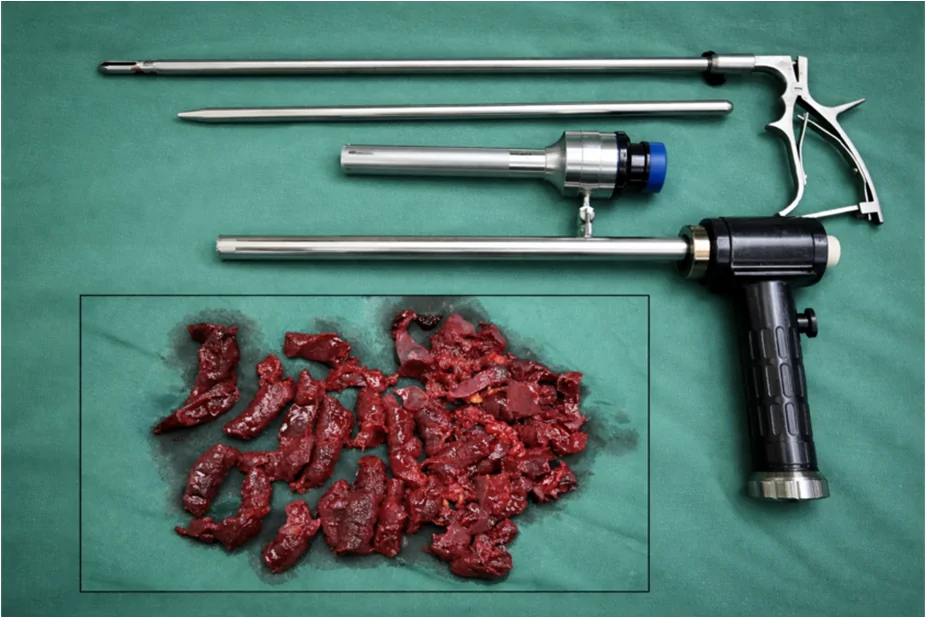
The power morcellator and extracted spleen tissue.

An electric power morcellator, equipped with a motor-driven cutting tube, was inserted into the CELIS system via Point E. A laparoscope was reinserted via Point A to provide intrabag illumination and a wide-angle field of view. Under direct vision, the morcellator was advanced layer by layer from the surface of the enlarged spleen towards its center, rapidly cutting the spleen into strip-like fragments that were directly suctioned extracorporeally (Figures 3 and 4). The entire rapid morcellation process was strictly confined within the transparent isolation bag, minimizing the risk of spillage.

#### 2.3.6 System removal (video 1)

(a) Upon completion of the morcellation, the sealing caps were reapplied to ensure the system was effectively airtight, and the main body of the system was returned to the abdominal cavity. (b) The gas within the bag was evacuated, and the conventional abdominal pneumoperitoneum was re-established. (c) The entire CELIS system, containing a small amount of residual fluid, was atraumatically extracted through the minimal incision at Point E.

#### 2.3.7 Irrigation and drainage

Following specimen removal, routine exploration was performed. The splenic fossa was irrigated with normal saline, and a drainage tube was placed.

#### 2.3.8 Final integrity testing

**Figure 5:**
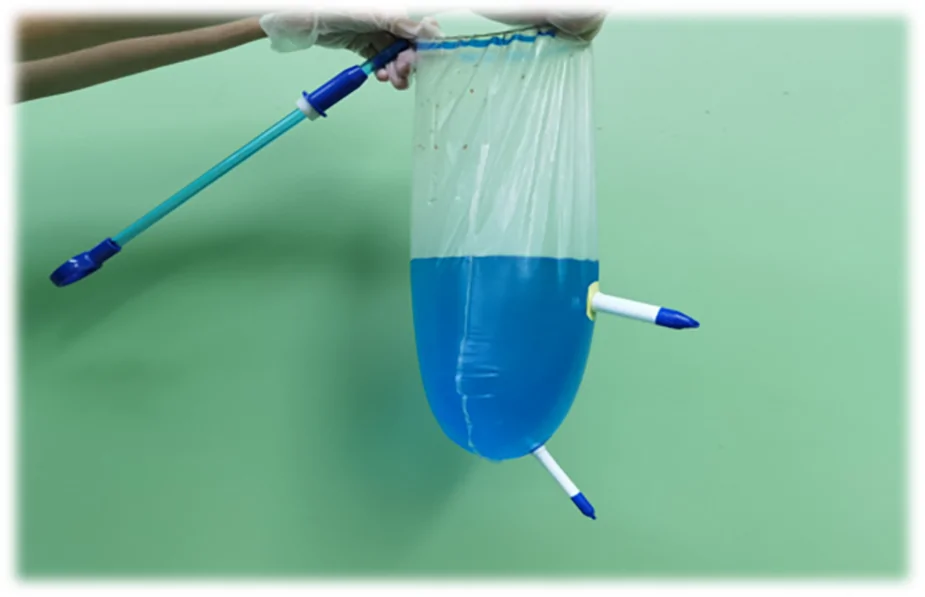
Identification of eventual system integrity using 1,000 mL of a methylene blue solution.

To assess system integrity, an integrity check of the system was mandatory after the procedure. To rigorously rule out any microscopic tears or leaks, 1,000 mL of methylene blue solution was injected under pressure into the extracted CELIS system (Figure 5). The preventive protocol dictated that if any sign of rupture was detected during the procedure, the operation must be immediately halted to either replace the system or convert to open surgery.

#### 2.3.9 Postoperative management

Regarding post-splenectomy care, all patients received perioperative vaccinations and asplenia-related management in strict accordance with the standardized institutional protocol.

### 2.4 Clinical characteristics and perioperative data collection

Data on the following clinical characteristics were collected retrospectively: age, sex, body mass index (BMI), diagnosis, and spleen volume index. The spleen volume index is a calculated surrogate parameter rather than a true 3D volumetric measurement; it is the product of the greatest superoinferior, anteroposterior, and mediolateral lengths measured at the level of the splenic hilum from images acquired using computed tomography or magnetic resonance imaging.

Core surgical data included total operative time, CELIS system setup and removal time, intrabag power morcellation time, estimated blood loss, and intraoperative adverse events (e.g., massive bleeding > 800 mL, collateral organ injury, or conversion to open surgery). Additionally, specific device-related complications (e.g., bag rupture) and postoperative complications were independently recorded. These included the length of hospital stay (LOS), 30-day readmission and reoperation rates, postoperative bleeding, wound complications, abdominal infection, pancreatic injury or fistula, portal/splenic vein thrombosis (monitored via routine postoperative ultrasound), pulmonary complications, and mortality. Postoperative complications were strictly graded according to the Clavien-Dindo classification system.

### 2.5 Subjective assessment metrics

Multidimensional scales were employed to accurately evaluate the subjective benefits to both patients and surgeons. Regarding postoperative analgesia, patients only received a standard dose of intravenous flurbiprofen axetil on postoperative day 1, without the routine use of opioids or patient-controlled analgesia (PCA) pumps. Postoperative pain was assessed on postoperative days 1 and 2 using the Visual Analog Scale (VAS, 0–10). Cosmetic satisfaction was evaluated at the 1-month outpatient follow-up using the Body Image Scale (BIS, lower scores indicate better outcomes) and the Cosmesis Scale (CS, higher scores indicate better outcomes) from the body image questionnaire. The operative workload of the surgeons was assessed immediately after each procedure by the two senior operating surgeons (Z.Y. and J.L.) using the Surgery Task Load Index (SURG-TLX), which subjectively measures the mental and physical demands of managing massive splenomegaly with the CELIS system across six dimensions. Each dimension was scored on a 0–10 scale, and the final reported value represents the unweighted average score (range: 0–10), with higher scores indicating greater workload.

### 2.6 Long-term follow-up

Patients were scheduled for follow-up outpatient visits at 1, 3, 6, and 12 months postoperatively, and annually thereafter. The median follow-up duration for the cohort was 61.5 months (range: 58–66 months). Routine follow-up included clinical examinations and abdominal ultrasound. Importantly, at the 5-year milestone, all 14 patients underwent both abdominal ultrasound and contrast-enhanced computed tomography to comprehensively evaluate for potential splenosis or ectopic splenic implantation. All imaging studies were retrospectively reviewed and interpreted by two independent senior radiologists who were blinded to the surgical techniques and clinical outcomes. Any discrepancies in image interpretation were resolved by consensus, and no evidence of ectopic spleen or splenosis was revealed.

### 2.7 Statistical analysis

Statistical analyses were performed in March 2026 using GraphPad Prism 10.0 software (Dotmatics, Boston, MA). Given that this is a single-arm retrospective cohort study designed to establish initial clinical data for a novel isolation system, data were primarily analyzed using descriptive statistics. Continuous variables were expressed as median (range) in addition to mean ± standard deviation (SD) to account for the small sample size; categorical variables and complication rates were expressed as frequencies (n) or percentages (%). For zero-event outcomes, 95% confidence intervals (CIs) were calculated using the exact binomial (Clopper-Pearson) method.

## 3 Results

The baseline clinical characteristics of the 14 patients are detailed in Table 1, while the detailed, patient-level clinical and pathological data are provided in Supplementary Table S1. The cohort comprised 9 males and 5 females, with a median age of 40.5 years (range: 24–60; mean: 41 ± 11.6) and a median BMI of 27.05 kg/m ^2^ (range: 21.5–30.8; mean: 26.67 ± 2.55). Notably, the cohort involved moderate to massive spleens, with a median splenic volume index of 1,488 cm^3^ (range: 780–3,000; mean: 1,727.07 ± 866.84). Preoperative diagnoses included splenic hemangioma (*n* = 10) and severe hypersplenism with enlarged splenomegaly secondary to cirrhotic portal hypertension (*n* = 4).

**Table 1:** Baseline clinical characteristics of the study cohort (*n* = 14).

Characteristics	Mea*n* ± SD/*n* (%)	Median, Range
Age, years	41.0 ± 11.6	40.5 (24–60)
Sex (Male/Female), *n* (%)	9 (64.3%)/5 (35.7%)	
BMI, kg/m ²	26.67 ± 2.55	27.05 (21.5–30.8)
Splenic Characteristics		
Main Indication, *n* (%)		
Symptomatic Hemangioma	10 (71.4%)	
Hypersplenism	4 (28.6%)	
Maximum Diameter, cm	18.46 ± 4.78	17.5 (13.0–25.5)
Spleen Weight, g	1,165 ± 614	980 (510–2,100)
Spleen Volume Index, cm³	1,727.07 ± 866.84	1,488 (780–3,000)
Surgical & Pathological Details		
Concomitant Procedures, *n* (%)		
None	9 (64.3%)	
Pericardial devascularization	4 (28.6%)	
Cholecystectomy	1 (7.1%)	
Final Pathology, *n* (%)		
Splenic hemangioma	10 (71.4%)	
Congestive splenomegaly	4 (28.6%)	

BMI, body mass index; SD, standard deviation.

Surgical metrics and follow-up outcomes are presented in Table 2. Despite the significant size of the organs, all 14 surgeries were successfully completed, with no cases requiring abdominal incision enlargement or conversion to open surgery. Intraoperative methylene blue testing confirmed that the CELIS system maintained a macroscopic physical integrity without detectable leakage. No collateral organ injuries occurred, and no blood transfusions were required within the entire cohort. As detailed in Table 3, the overall postoperative recovery was uneventful, with a mean length of hospital stay of 5.1 ± 1.1 days. According to the Clavien-Dindo classification, two patients experienced minor Grade I complications: one patient (Patient 3) had mild postoperative nausea requiring only routine antiemetics, and one patient (Patient 7) developed a transient mild fever managed with standard antipyretics. No patients experienced major complications (Grade II or above). Specifically, there were no recorded instances of postoperative bleeding, wound complications, abdominal infection, pancreatic fistula, portal or splenic vein thrombosis, pulmonary complications, readmission, reoperation, or mortality (0/14).

**Table 2:** Surgical outcomes (*n* = 14).

Variables	Mean ± SD	Median (Range)
Operative time, min	182.86 ± 52.84	185 (110–275)
Placement time, min	5.50 ± 1.77	5 (3–9)
Removal time, min	3.79 ± 1.00	3.5 (2–6)
Morcellation time, min	20.71 ± 2.12	20.5 (17–25)
Estimated blood loss, mL	160.71 ± 134.88	120 (50–500)
Device-related complications *n* (%)	0/14 (95% CI: 0.0% – 23.2%)	–
VAS score^a^		
D1 after surgery	2.64 ± 0.61	2.5 (2–4)
D2 after surgery	2.21 ± 0.41	2.0 (1.5–3)
Body image score^b^	6.71 ± 1.10	6.5 (5–9)
Cosmesis score^c^	21.36 ± 0.97	21.5 (19–23)
SURG-TLX^d^	3.43 ± 0.82	3.5 (2–5)

a VAS, visual analogue scale (0 indicates no pain, whereas 10 represents excruciating pain).

b Body image score (score range: between 5 and 20, with a low score meaning a better body image).

c Cosmesis score (score range: between 3 and 24, with a high score indicating better cosmetic results).

d SURG-TLX, the surgery task load index (scored on a 0–10 scale, with a higher score representing more cognitive/physical load in surgery. The reported value is the average of the six dimensions). SD, standard deviation.

**Table 3:** Detailed postoperative outcomes and complications of the 14 patients.

Patients	LOS (days)	Clavien-Dindo grade	Postoperative bleeding	Wound complication	Pancreatic fistula/infection	Portal	Splenic vein thrombosis	Pulmonary complication	30-day readmission / reoperation
1♂	5	None	None	None	None	None	None	None	None
2♂	6	None	None	None	None	None	None	None	None
3♀	4	Ⅰ^a^	None	None	None	None	None	None	None
4♀	4	None	None	None	None	None	None	None	None
5♂	7	None	None	None	None	None	None	None	None
6♀	6	None	None	None	None	None	None	None	None
7♀	5	Ⅰ^a^	None	None	None	None	None	None	None
8♂	4	None	None	None	None	None	None	None	None
9♂	4	None	None	None	None	None	None	None	None
10♂	4	None	None	None	None	None	None	None	None
11♂	4	None	None	None	None	None	None	None	None
12♂	6	None	None	None	None	None	None	None	None
13♂	7	None	None	None	None	None	None	None	None
14♀	5	None	None	None	None	None	None	None	None
Mean ± SD	5.1 ± 1.1	--	--	--	--	--	--	--	--
Median (Range)	5 (4–7)	--	--	--	--	--	--	--	--

a Two patients experienced Clavien-Dindo grade I postoperative complications: Patient 3 had mild postoperative nausea requiring routine antiemetics, and Patient 7 developed a transient mild fever managed with standard antipyretics.

The median total operative time was 185 min (range: 110–275; mean: 182.86 ± 52.84). The operation of the CELIS system demonstrated feasibility: the median setup time was 5 min (range: 3–9; mean: 5.5 ± 1.77), and the median removal time was 3.5 min (range: 2–6; mean: 3.79 ± 1.01). The median time for complete morcellation and extraction was 20.5 min (range: 17–25; mean: 20.71 ± 2.12).

Although postoperative recovery was generally favorable, five patients in this cohort underwent concomitant procedures (pericardial devascularization, *n* = 4; cholecystectomy, *n* = 1). Consequently, global perioperative metrics, such as total operative time, estimated blood loss, length of hospital stay, and subjective pain and recovery scores, reflect the cumulative surgical burden. These outcomes cannot be attributed solely to the CELIS-assisted procedure. The median VAS score was 2.5 (range: 2–4; mean: 2.64 ± 0.61) on postoperative day 1. At the 1-month follow-up, the median BIS score was 6.5 (range: 5–9; mean: 6.71 ± 1.10), and the median CS score was 21.5 (range: 19–23; mean: 21.36 ± 0.97). Furthermore, the median SURG-TLX score was 3.5 (range: 2–5; mean: 3.43 ± 0.82), indicating acceptable physical and mental demands on the surgeons during this novel procedure.

### 3.1 5-Year follow-up results

Following the 5-year clinical follow-up with ultrasound and contrast-enhanced CT, all 14 patients were alive and in good health without any detected abnormalities (Supplementary Table S1). Notably, within this selected preliminary cohort, no clinically or radiologically detectable splenic tissue implantation (splenosis) or ectopic splenic implantation was observed in the abdominal cavity, pelvic cavity, or trocar port sites (0/14, 0%; 95% CI: 0.0%–23.2%). This provides preliminary observational support of its containment capabilities in this limited sample.

## 4 Discussion

Over the past century, the evolution of surgery has been fundamentally driven by the pursuit of minimizing iatrogenic trauma. The widespread adoption of LS has transformed it into the gold standard within the realm of minimally invasive surgery (9). However, despite continuous advancements in surgical techniques, specimen retrieval remains a major bottleneck when managing massive splenomegaly. The clinical paradox of “resectable but not retrievable” continues to be a critical hurdle in current practice.

Traditionally, enlarging the existing abdominal incision or creating a new laparotomy has been employed to facilitate specimen extraction. However, this approach inherently compromises the core advantages of minimally invasive surgery. Previous studies comparing laparoscopic and open splenectomies have demonstrated that the minimally invasive approach is strongly associated with reduced postoperative pain, shorter hospital stays, and fewer wound-related complications, underscoring the importance of avoiding incision enlargement whenever possible (9). Although posterior colpotomy offers an effective alternative for specimen retrieval, its application is precluded in male patients and nulliparous women, and it presents extreme technical difficulties in patients with vaginal stenosis or extraordinarily large spleens (10).

A distinct advantage of laparoscopic splenectomy is that retrieving an intact spleen is generally unnecessary for adequate pathological analysis in benign or non-malignant hematological conditions (11). In our cohort, the power morcellator extracted the spleen in strip-like fragments that sufficiently preserved the macroscopic and microscopic tissue architecture. Consequently, morcellation did not limit or compromise the final pathological evaluation in any case, and all preoperative benign diagnoses were successfully confirmed postoperatively. This allows for the morcellation of the spleen and subsequent extraction through a smaller incision.

While power morcellation effectively resolves the extraction bottleneck, unprotected tissue morcellation introduces a severe biological hazard: intraperitoneal tissue dissemination. The risk of splenosis—defined as the ectopic autotransplantation of splenic tissue—following laparoscopic splenectomy should not be underestimated (4). Although uncommon, experimental and clinical evidence indicates that viable splenic tissue may implant and proliferate within the abdominal or pelvic cavity after intraoperative fragmentation or specimen retrieval failure (5). For instance, Lansdale reported recurrent thrombocytopenia caused by intra-abdominal splenosis after laparoscopic splenectomy in a child in whom the retrieval bag ruptured during morcellation (12). Similarly, Sato described splenosis following laparoscopic splenectomy, highlighting the potential consequences of splenic tissue spillage (13).

Beyond recurrent hematological symptoms, splenosis can manifest with non-specific abdominal pain or bowel obstruction (14). More importantly, its radiological presentation frequently mimics peritoneal carcinomatosis or lymphadenopathy, leading to misdiagnosis and prompting unnecessary invasive interventions (15). These observations suggest that even minimally invasive approaches do not inherently eliminate the risk of tissue dissemination, emphasizing the critical imperative for reliable containment systems during specimen extraction.

The concept of contained morcellation has gained increasing attention in minimally invasive surgery following concerns regarding tissue dissemination associated with open power morcellation. Cohen et al. first described morcellation within an insufflated isolation bag, demonstrating the technical feasibility of complete tissue containment (16). Subsequent studies comparing contained and uncontained morcellation confirmed that in-bag techniques can reduce the theoretical risk of tissue dissemination while preserving the advantages of minimally invasive surgery. The CELIS system extends this containment concept to laparoscopic splenectomy and massive splenic specimens, which present unique challenges because of their large size, friability, and regenerative potential.

To address this dilemma, the CELIS system offers a comprehensive solution. Unlike conventional specimen retrieval bags, the CELIS system establishes a completely enclosed working environment. Briefly, after the spleen is detached and placed into the system, the abdominal cavity is desufflated, and the interior of the system is simultaneously insufflated to establish an “intrabag pneumoperitoneum.” This technical modification creates a stable and expanded operative space, overcoming the spatial constraints imposed by massive specimens. This design minimizes the mechanical stress exerted on the isolation system, thereby potentially mitigating the risk of bag rupture.

Furthermore, the system is equipped with a multi-port rigid trocar that serves as an auxiliary port, enabling multi-channel operations under direct visualization. The simultaneous use of a laparoscope and a power morcellator ensures that the tissue fragmentation process is entirely controllable and precise. Compared with traditional blind or semi-blind techniques, this may facilitate the morcellation process. Concurrently, the system's sealing cap ensures that the internal splenic tissue is completely encapsulated, avoiding direct contact with the abdominal wall incision during extraction.

Although preliminary, our results corroborate these theoretical advantages. Regarding the subjective assessments, the favorable VAS, BIS, and CS scores reflect adequate postoperative pain control and patient satisfaction with the preserved abdominal cosmesis. Moreover, the SURG-TLX results indicate that the cognitive and physical demands of in-bag morcellation remain within a manageable threshold for the operating surgeons. Importantly, given the absence of a comparative control group, these metrics function strictly as descriptive tools; they serve to confirm the clinical tolerability of the CELIS device rather than to demonstrate its superiority over conventional extraction techniques. During the postoperative follow-up period, no instances of system rupture, surgical complications, or splenosis were observed. For our initial case, the deployment and extraction times for the CELIS system were 10 and 6 min, respectively, with a morcellation time of 25 min. As surgeons navigate the learning curve and gain proficiency with the system and power morcellation, the entire specimen retrieval process can be routinely completed within 30 min.

Notably, our 5-year follow-up outcomes provide preliminary support for this approach. Within this selected preliminary cohort, not a single case of splenosis or intraperitoneal dissemination was observed. Given that splenosis is a well-documented long-term sequela of splenic tissue spillage, this finding suggests the potential value of the “completely contained isolation” concept, although the absence of events in 14 patients does not exclude a clinically relevant risk.

Beyond splenectomy, the paradigm of fully enclosed specimen management holds broader translational potential. In other surgical disciplines where tissue dissemination poses a risk (e.g., laparoscopic myomectomy in gynecology), platforms like CELIS that establish a hermetic barrier could offer a universal solution to elevate the overall safety of minimally invasive surgery.

It is important to acknowledge that the methylene blue test used in our study only verifies the macroscopic physical integrity of the CELIS bag, rather than providing microscopic cytological endpoints (such as peritoneal washing cytology). However, the formation of splenosis typically requires a prolonged latency period to become clinically or radiologically evident. In this context, our 5-year clinical follow-up with ultrasound and contrast-enhanced CT did not identify clinically or radiologically detectable splenosis.

## 5 Limitations

This study is not without limitations. First, it is a single-center retrospective study with a very small sample size (*n* = 14) and lacks a direct comparative control group, which inherently introduces selection bias. The cohort may primarily comprise cases with favorable splenic anatomy selected during our initial learning curve. Second, performing power morcellation within a confined, flexible pneumo-bag environment demands advanced hand-eye coordination and a definitive learning curve. Surgeons must adapt to restricted instrument angles and visual feedback to achieve the rapid morcellation efficiency reported in this cohort. Third, the routine application of the CELIS device inevitably increases the overall equipment cost per procedure. Future large-scale, multicenter randomized controlled trials are mandatory to definitively evaluate its cost-effectiveness, establish standardized training protocols, and confirm its generalizability in broader clinical settings. Fourth, while no clinical or radiological evidence of splenosis was observed in our cohort, conventional imaging modalities such as ultrasound and contrast-enhanced CT have limited sensitivity for detecting small, microscopic, or clinically silent splenic implants. Furthermore, highly sensitive nuclear medicine imaging techniques, such as heat-damaged red blood cell spleen scintigraphy or SPECT/CT, which are considered the gold standard for detecting residual splenic tissue, were not utilized in our follow-up protocol. Therefore, the presence of subclinical micro-splenosis cannot be definitively ruled out. Fifth, the inclusion of concomitant procedures (e.g., pericardial devascularization, cholecystectomy) in a subset of patients inherently confounds the general perioperative outcomes, as the reported total operative time, blood loss, and pain scores reflect the combined burden of all surgeries rather than the isolated impact of CELIS-assisted splenectomy. However, this did not affect the evaluation of aspects such as CELIS setup and morcellation times, as well as the 5-year absence of splenosis. Finally, the subjective assessment tools used (VAS, BIS, CS, SURG-TLX), while widely accepted, have not been validated specifically for this novel setting. Given the absence of a control group, these metrics serve strictly to document clinical tolerability rather than establishing superiority over conventional extraction methods.

## 6 Conclusion

In conclusion, the CELIS system offers an approach compatible with conventional laparoscopy that enables fully enclosed in-bag splenic morcellation, potentially mitigating the risk of splenosis and ectopic splenic implantation. Observations from this small cohort indicate that the system is a feasible technical adjunct for contained specimen extraction, avoiding the need for incision enlargement during laparoscopic splenectomy. However, these preliminary findings are based on a limited, uncontrolled sample. Future large-scale, prospective comparative studies are required to rigorously evaluate its clinical efficacy and long-term outcomes before broader clinical adoption can be recommended.

## Data Availability

The original contributions presented in the study are included in the article/supplementary Material, further inquiries can be directed to the corresponding authors.
