# Supplementary material for: Contained morcellation of the spleen using a completely enclosed laparoscopic isolation system: a 5-year follow-up study on the evaluation of splenosis
Source: Front Surg. 2026 Aug 11;13:1876074. doi: 10.3389/fsurg.2026.1876074 (PMC13504647; doi:10.3389/fsurg.2026.1876074)
Supplement: Supplementary file 1 [file Datasheet1.pdf]

Supplementary Material

1 Supplementary Data

Supplementary Video 1: Demonstration of the CELIS-assisted laparoscopic splenectomy. The video highlights the core surgical procedures, including the insertion of the CELIS system, establishment of the intrabag pneumoperitoneum, fully closed direct-vision power morcellation of the massive spleen, and the atraumatic removal of the system.

Supplementary table 1 Patient clinical characteristics.

Patients	Age	BMI, kg/m^2	Main Indication	Maximum Diameter (cm)	Spleen Weight (g)	Spleen volume index(cm^3)	Other procedures	Final Pathology	Follow-up Outcomes
1♂	31	30.8	Symptomatic Hemangioma	16.5	880	1344	Cholecystectomy	Splenic hemangioma	NAD
2♂	52	25.3	Hypersplenism	21.0	1450	2142	Pericardial devascularization	Congestive splenomegaly	NAD
3♀	28	21.5	Symptomatic Hemangioma	14.0	960	960	No	Splenic hemangioma	NAD
4♀	35	27.7	Symptomatic Hemangioma	14.5	990	990	No	Splenic hemangioma	NAD
5♂	54	24.5	Hypersplenism	25.5	2100	3000	Pericardial devascularization	Congestive splenomegaly	NAD
6♀	54	27.3	Symptomatic Hemangioma	25.0	2050	3000	No	Splenic hemangioma	NAD
7♀	60	28.3	Symptomatic Hemangioma	18.5	1080	1632	No	Splenic hemangioma	NAD
8♂	30	29.9	Symptomatic Hemangioma	15.0	730	1120	No	Splenic hemangioma	NAD
9♂	50	30.0	Symptomatic Hemangioma	13.5	550	825	No	Splenic hemangioma	NAD
10♂	28	23.7	Symptomatic Hemangioma	13.0	510	780	No	Splenic hemangioma	NAD

11♂	24	24.7	Symptomatic Hemangioma	13.5	560	840	No	Splenic hemangioma	NAD
12♂	47	26.8	Hypersplenism	24.5	1950	2880	Pericardial devascularization	Congestive splenomegaly	NAD
13♂	46	27.5	Hypersplenism	25.0	2080	3000	Pericardial devascularization	Congestive splenomegaly	NAD
14♀	35	25.4	Symptomatic Hemangioma	19.0	1100	1666	No	Splenic hemangioma	NAD
Mean± SD	41±11.60	26.67±2.5	--	18.46±4.78	1165±614	1727.07±866.84	--	--	--
Median (Range)	40.5 (24 – 60)	27.05 (21.5 – 30.8)	--	17.5 (13.0 – 25.5)	980 (510 – 2100)	1488 (780 – 3000)	--	--	--

BMI: body mass index, SD: standard deviation,NAD: No abnormalities detected.
